# PTEN Expression Regulates Gap Junction Connectivity in the Retina

**DOI:** 10.3389/fnana.2021.629244

**Published:** 2021-05-20

**Authors:** Ashley M. Chen, Shaghauyegh S. Azar, Alexander Harris, Nicholas C. Brecha, Luis Pérez de Sevilla Müller

**Affiliations:** ^1^Department of Neurobiology, David Geffen School of Medicine at Los Angeles, University of California, Los Angeles, Los Angeles, CA, United States; ^2^Stein Eye Institute, David Geffen School of Medicine at Los Angeles, University of California, Los Angeles, Los Angeles, CA, United States; ^3^CURE Digestive Diseases Research Center, David Geffen School of Medicine at Los Angeles, University of California, Los Angeles, Los Angeles, CA, United States; ^4^Veterans Administration Greater Los Angeles Health System, Los Angeles, CA, United States

**Keywords:** connexin (Cx36), PTEN (phosphatase and tensin homolog deleted on chromosome 10), retinal ganglion cells, degeneration, retina, gap junction (connexin)

## Abstract

Manipulation of the phosphatase and tensin homolog (PTEN) pathway has been suggested as a therapeutic approach to treat or prevent vision loss due to retinal disease. In this study, we investigated the effects of deleting one copy of *Pten* in a well-characterized class of retinal ganglion cells called α-ganglion cells in the mouse retina. In *Pten*^+/–^ retinas, α-ganglion cells did not exhibit major changes in their dendritic structure, although most cells developed a few, unusual loop-forming dendrites. By contrast, α-ganglion cells exhibited a significant decrease in heterologous and homologous gap junction mediated cell coupling with other retinal ganglion and amacrine cells. Additionally, the majority of OFF α-ganglion cells (12/18 cells) formed novel coupling to displaced amacrine cells. The number of connexin36 puncta, the predominant connexin that mediates gap junction communication at electrical synapses, was decreased by at least 50% on OFF α-ganglion cells. Reduced and incorrect gap junction connectivity of α-ganglion cells will affect their functional properties and alter visual image processing in the retina. The anomalous connectivity of retinal ganglion cells would potentially limit future therapeutic approaches involving manipulation of the Pten pathway for treating ganglion cell degeneration in diseases like glaucoma, traumatic brain injury, Parkinson’s, and Alzheimer’s diseases.

## Introduction

The phosphatase and tensin homolog deleted on chromosome ten (Pten) protein is a potential molecular target for novel therapeutic strategies to treat optic neuropathies (Guzman-Aranguez et al., 2013; Ogino et al., 2016). Down-regulation of Pten activates the mTOR pathway (Jaworski et al., 2005; Park et al., 2008; Kim et al., 2009; Zukor et al., 2013), resulting in optic nerve axonal regeneration and a concomitant increase in the number of surviving retinal ganglion cells (RGCs) after nerve injury (Park et al., 2008; Leibinger et al., 2012; de Lima et al., 2012; Duan et al., 2015). Paradoxically, in uninjured retinas, Pten deletion at the onset of neurogenesis in retinal progenitor cells or in a conditional *Pten* loss-of-function allele, results in reduced number of RGCs and rod photoreceptors, and disrupts amacrine cell dendritic arborizations (Cantrup et al., 2012; Jo et al., 2012; Sakagami et al., 2012; Tachibana et al., 2016). In cell cultures, silencing *Pten* with non-targeting small-interfering RNA (NT-siRNA) decreases gap junctional intercellular communication between glia cells (González-Sánchez et al., 2016), perhaps due to Pten’s effect on protein kinases (Preiss et al., 2007), which mediate phosphorylation of connexins (Xia and Mills, 2004; Urschel et al., 2006; Pérez de Sevilla Müller et al., 2010a).

Gap junctions are intercellular channels formed by connexins between retinal neurons that influence the propagation and integration of visual signals (Bloomfield and Völgyi, 2009). Gap junctions are reported to participate in neuronal spike synchrony to enhance the saliency of visual signals, and mediate changes in light adaptation and circadian rhythms (Söhl et al., 2005; Hartveit and Veruki, 2012; Völgyi et al., 2013a; O’Brien, 2014). Gap junctions have also been linked to a number of neurological pathologies (Nakase and Nasus, 2004) as they might allow the passing of toxic molecules from dying cells to neighboring healthy cells (Krysko et al., 2005; Rodríguez-Sinovas et al., 2007). For example, blockade of Cx36 gap junctions provides RGC protection in glaucoma models (Akopian et al., 2014, 2016; Chen et al., 2015).

With downregulation of Pten as a potential target to increase axonal growth and enhance RGC survival in retinal diseases (Park et al., 2008; Leibinger et al., 2012; de Lima et al., 2012; Duan et al., 2015), understanding the fundamental roles that *Pten* plays in forming and maintaining RGC architecture and connectivity is of high importance, which will impact future clinical therapies that manipulate *Pten* signaling.

To selectively study *Pten* signaling on RGCs, we used loxP-mediated recombination to generate mice in which parvalbumin (PV) cells lacked one copy of *Pten* (Baohan et al., 2016). In the present study, we evaluate the effect of *Pten* loss on PV-RGCs, focusing on α-RGC architecture and gap junction connectivity. Our data revealed that the lack of one copy of *Pten* does not alter the morphology of α-RGCs. However, ON and OFF α-RGCs exhibited a significant decrease of coupled cells or were uncoupled. Additionally, we observed that the majority of OFF α-RGCs lost their normal coupling patterns but showed novel coupling to displaced amacrine cells. The number of connexin36 puncta in OFF α-RGCs was decreased by at least 50% when compared to control OFF α-RGCs.

## Materials and Methods

These studies were conducted under protocols approved by the University of California at Los Angeles (UCLA) Animal Research Committee. All experiments were carried out in accordance with guidelines for the welfare of experimental animals issued by the U.S. Public Health Service Policy on Human Care and Use of Laboratory Animals, and the UCLA Animal Research Committee.

### Animals and Animal Preparation

*PV-Cre^+/^*^−^*/Ai9^+/^*^−^*/Pten^loxP+/^*^−^*^mice^*: Animals were provided by Dr. J. Trachtenberg at UCLA (Baohan et al., 2016). The generation of mice carrying a conditional *Pten* loxP/loxP allele flanking exon 5, which encodes PTEN’s phosphatase domain, has been described previously (Lesche et al., 2002). To genetically label PV-positive neurons, PV-IRES-cre knock-in female mice (Jackson Laboratories, stock#008069, Hippenmeyer et al., 2005) were crossed with male tdTomato reporter knockin mice (Jackson Laboratories, stock #007905, “Ai9”, Madisen et al., 2010). Offspring were hemizygous for both transgenes. These mice were then crossed with PV-Cre mice to generate offspring that were homozygous for PV-Cre and hemizygous for Ai9. Hemizygous mice for both Ai9 and a floxed *Pten* were generated using separate breeders (Lesche et al., 2002). PV-Cre^+/+^/Ai9^+/–^ mice were then crossed with Ai9^+/–^/*Pten*^loxP+/–^ mice to generate offspring for Cre, Ai9 and *Pten* in PV cells (Baohan et al., 2016). Littermate controls were hemizygous for Cre and Ai9 in PV cells, but expressed both copies of *Pten*. Only the hemizygous animals (PV-*Pten*^+/–^) were studied, as complete knockouts died prematurely from seizures (Baohan et al., 2016) or developed untreatable head tilt and impaired mobility/neuropathy.

Both male and female mice were used for these studies. Animals were 3–5 months old at the time of experimentation. Following deep anesthesia with 1%-3% isoflurane (Abbott Laboratories, North Chicago, IL, USA), animals were euthanized by cervical dislocation. The eyes were enucleated and dissected in Hibernate A (Invitrogen, Carlsbad, CA) or Hank’s Balanced Salt Solution (HBSS) (ThermoScientific, Waltham, MA) on ice for fluorescence and immunohistochemical studies, and in bicarbonate-buffered Ames medium (pH 7.4) at room temperature (RT) for the intracellular dye injection studies.

### Immunohistochemistry

Immunohistochemical labeling was performed using our published protocols (Pérez de Sevilla Müller et al., 2007, 2010a,b, 2013, 2017, 2019). Whole-mounted retinas were fixed for 15 min by immersion in 4% paraformaldehyde (PFA) in 0.1 M PB (pH 7.4) at RT. They were subsequently washed in phosphate buffer (PB) three times for a total of 90 min and incubated in 10% normal goat serum (NGS) with 0.3%–0.5% Triton X-100 at 4°C overnight. Retinas were incubated in primary antibodies (Table 1) for 7 days at 4°C. They were then rinsed three times for 30 min each with 0.1 M PB and incubated with the corresponding secondary antibodies overnight at 4°C. The following day, the retinas were washed three times in 0.1 M PB and mounted in Vectashield mounting medium (Vector Laboratories, Burlingame, CA, USA), Citifluor (Citifluor, London, UK) or Aqua Poly-Mount (Polysciences, Warrington, PA, USA). Control experiments for nonspecific binding of the secondary antibodies were also performed.

**Table 1:** Antibodies used in this study.

Antibody	Host	Immunogen	Source	Dilution
Calretinin	Mouse	Recombinant human calretinin-22k	Swant; Bellinzona, Switerzerland; Lot no. 010399 clone 6B3.	1:2,000
ChAT	Goat	Human placental choline acetyltransferase.	Millipore; Temecula, CA, USA; AB144P	1:500
Cx36	Mouse	Connexin36 C-terminal region of rat and mouse Cx36 (aa 286–303).	Zymed; South San Francisco, CA; 37-4600.	1:500
Glutamic Acid Decarboxylase 67 (GAD_67_)	Mouse	Amino acid residues 4–101 of human GAD67.	EMD Millipore; Temecula, CA, USA; MAB5406, AB_2278725.	1:1,000
Glycine	Rat	Glycine conjugated to paraformaldehyde and carrier protein thyroglobulin.	ImmunoSolution; Everton Park, QLD, Australia, IG1002.	1:1,000
Goα	Mouse	Bovine brain Go-alpha purified	Millipore; Temecula, CA, USA; MAD3073	1:300
PKC	Mouse	Purified bovine PKC and its epitope mapped to its hinge region (amino acids 296–317).	Biodesign International ME K01107M.	1:1,000
Prox1	Rabbit	C-terminal 15 amino acids of mouse Prox1	BioLegend; San Diego, CA, USA PRB-238C.	1:1,000–1:2,000
RNA binding protein multiple splice (RBPMS)	Guinea pig	N-terminus of the RBPMS polypeptide (RBPMS_4–24_) GGKAEKENTPSEANLQEEEVR	PhosphoSolutions, Colorado, USA 1832-RBPMS, AB_10890167.	1:1,000
Vesicular glutamate transporter 1 (VGluT1)	Guinea pig	Amino acid residues 541–560 of rat VGluT1.	EMD Millipore; Temecula, CA, USA; AB5905, AB_2301751.	1:1,000

To prepare retinal sections, eyecups were submerged in 4% PFA in 0.1 M PB, pH 7.4, for 15, 30, 45, or 60 min at RT. They were then placed in 30% sucrose in PB overnight at 4°C. The eyecups were embedded in optimal cutting temperature medium (Sakura Finetek, Torrance, CA, USA) and sectioned at 12–14 μm with a Leica CM3050S (Leica Microsystems, Buffalo Grove, IL, USA).

Sections were washed three times in 0.1 M PB and incubated in a solution of 10% NGS or donkey serum (DS), 1% bovine serum albumin (BSA), and 0.3–0.5% Triton X-100 in 0.1 M PB for 1–2 h at RT. Following removal of the blocking solution, the slides were immediately incubated in the primary antibodies (Table 1) diluted in PB with 0.3–0.5% Triton X-100 and 0.1% NaN_3_, overnight at 4°C. Retinal sections were washed three times for a total of 30 min with 0.1 M PB, and the corresponding secondary antibodies (1:1,000; Invitrogen, Carlsbad, CA) were then applied for 1–2 h at RT in the dark. The tissues were washed three times for 10 min each in 0.1 M PB, and the retinal sections were then coverslipped with Aqua Poly/Mount (Polysciences, Inc., Warrington, PA), Vectashield (Vecta Laboratories), or Citifluor (Citifluor, London, UK).

The dilutions of the primary antibodies are given in Table 1. Secondary antibodies used in this study were Alexa-488 goat anti-guinea pig IgG, Alexa-488 goat anti-mouse IgG, Alexa-488 goat anti-rat IgG, Alexa-488 or −568 goat anti-rabbit IgG, and Alexa-488 donkey anti-goat IgG. As a negative control, the omission of the primary antibodies in the single or double labeling studies confirmed the elimination of nonspecific labeling.

All antibodies employed in this study have been used previously with PFA-fixed tissue; our immunostaining patterns were identical to those previously reported in studies using mouse retina (Haverkamp and Wässle, 2000; Pérez de Sevilla Müller et al., 2013; Rodriguez et al., 2014).

### Intracellular Dye Injections Studies

Intracellular injections were carried out from 1 pm to 5 pm. The retina was flattened with four radial cuts and mounted with the photoreceptor side down on black filter article. The tissue was then transferred to a bicarbonate-buffered Ames medium (pH 7.4) that was bubbled continuously with carbogen (95% O_2_/5% CO_2_) and mounted on a Zeiss Axioskop 2.

Intracellular injections of Lucifer Yellow and Neurobiotin were performed as described earlier (Pérez de Sevilla Müller et al., 2007, 2010a,b; Vuong et al., 2015). Borosilicate glass electrodes (#60200; A-M Systems; Sequim, WA, USA) were pulled and filled at their tips with 0.5% Lucifer Yellow (Sigma–Aldrich) and 4% N-(2-aminoethyl)-biotinamide hydrochloride (Neurobiotin; Vector Laboratories, Burlingame, CA, USA), and back-filled with 0.1 M Tris buffer, pH 7.4. TdTomato fluorescent cell bodies in the GCL were visualized with a Zeiss long-working distance 40× water immersion objective and conventional epifluorescence for Cy3. Lucifer Yellow was iontophoresed with negative current of −1 nA. When the morphology of the ganglion cell was revealed, the polarity of the current was reversed (+1 nA) and Neurobiotin was injected for 3 min. Multiple cells were injected in each retina, with 1–3 injected α-RGCs in each quadrant. After the final injection, the retina was left in the chamber for at least 30 min to allow for the tracer to diffuse into the cells. Retinas were then fixed in 4% PFA for 15 min and washed for 30 min in 0.1 M PB. To visualize Neurobiotin, retinas were incubated overnight with streptavidin–fluorescein (FITC; dilution 1:500; Jackson Immunoresearch, West Grove, PA), in 0.1 M PB containing 0.3% Triton X-100 at 4°C. On some occasions, streptavidin–fluorescein immunolabeled mis-injected retinal Müller cells close to the Neurobiotin-injected ganglion cell body. Müller cells were not included in the coupling analysis. Retinas were mounted with the GCL facing upward and cover slipped with Aqua Poly/Mount.

### Fluorescent Image Acquisition

Labeling was assessed with a Zeiss laser scanning microscope 710 or 880 (Zeiss LSM 710/Zeiss LSM 880; Carl Zeiss, Thornwood, NY, USA; RRID: SciEx_11637) with a Zeiss C-Apochromat 40× 1.2NA C-Apochromat corrected water objective or Zeiss C-Aprochromat 63× /1.4 corrected oil objective. The images were captured at a resolution of 512 × 512, 1,024 × 1,024 or 2,048 × 2,048 pixels. Images are presented as projection images of 3–130 image scans (*z*-axis step ranged from 0.3 to 1 μm).

### Analysis of Whole-Mounted Retinas

Retinal whole-mounts were imaged in their entirety as tiled mosaics and stitched together with a 5–10% overlap at the edge of each optical section, using the Zeiss Zen 2011 Black software (version 3.2) package. Individual tiles were collected as 12-bit 3D z-stacks from the nerve fiber layer to the INL using a 40× /1.20NA C-Apochromat objective and a zoom factor of 1. The confocal pinhole diameter was set to 1 Airy unit and pixel acquisition set to 1024 × 1024. Cells in retinal fields (500 × 500 μm or 1000 × 1000 μm) at 100 μm intervals from the optic nerve head to peripheral retina were manually counted. Three retinal fields per quadrant of each retina were analyzed for PV-cells and Cx36 counts.

### Analysis of Retinal Sections

Retinal sections were imaged in their entirety as tiled mosaics and stitched together with a 5–10% overlap at the edge of each optical section, using the Zeiss Zen 2011 Black software (version 3.2) package using a Zeiss C-Apochromat 40 × /1.2 NA corrected water objective and a zoom factor of 0.6. The confocal pinhole diameter was set to 1 Airy unit and pixel acquisition set to 512 × 512. Cells in areas of 500 μm were manually counted. At least four retinal fields of each section were analyzed.

### Analysis of Injected Ganglion Cells

Confocal images were analyzed using the Zeiss proprietary software (version 3.2), Image Browser v4 or Imaris 9.5.0 (Bitplane AG, Concord, MA, USA) software.

RGCs were reconstructed using the Imaris Filament Tracer option. The Filament Tracer operates on 3D images, which provides sufficient resolution to resolve the Filaments to be studied in all three spatial directions. The Filament Tracer option automatically computes all the paths from a user-defined starting point (RGC body) to the end of the structure. After all possible paths are calculated by the algorithm, the filaments are traced by the user by moving the mouse over the structure of interest. Imaris provided the following morphological analysis:

Dendrite Length: the sum of all edges between two branch points or between a branch point and a terminal point, respectively.

Filament Volume (sum): the sum of all segment’s volumes within the entire filament graph.

Filament-Dendrite Area: defined as the sum of the areas of all the segment edges. The area of an edge is defined as a surface area of a frustum (truncated cone).

Sholl Analysis: the number of dendrite intersections on concentric spheres, defining dendrite spatial distribution as a function of distance from the beginning point.

Number of Dendrite Branch Points: the number of dendrite branching points in the entire filament graph.

Filament-Dendrite Straightness: the ratio between dendrite length and radial distance between two branch points. If the Dendrite Straightness is 1 that means that the dendrite is completely straight.

Filaments-Dendrite Branching Angle B: the angle between the extending lines connecting the branch point with the neighboring branch points and the terminal points, respectively.

### Analysis of Cx36 in Injected Ganglion Cells

For quantitative analysis of RGC morphology and Cx36 synaptic puncta associated with their dendrites, individual optical slices from Z-stacks were analyzed using the Imaris software. Injected RGCs were reconstructed using the Imaris Filament Tracer option to create a 3D cell-surface rendering using a combination of surface and filament objects as described above. RGCs were masked using an automated threshold determined by the software.

Cx36 puncta were first reconstructed as 3D structures using “surface objects” (to outline puncta borders) created using an estimated 0.5 μm diameter. Spots were created (using software-determined automatic threshold) for all synaptic puncta. Puncta “objects” were then converted into puncta “spots” (with automatic intensity max spot detection thresholds and a 0.5 μm estimated diameter) using surface object centroids in Imaris. All spots located less than 0.3 μm from the surface of the RGC mask were quantified. The intensity levels and contrast of the final images presented in the figures were adjusted in Adobe Photoshop CS2 v.9.02 (Adobe Systems, San Jose, CA).

### Statistical Analysis

All values are given as mean and standard error of the mean (SEM). Single statistical comparisons of a group vs. its control group were performed using a two-tailed Student’s t-test in GraphPad Prism 4.0 (GraphPad Software, Inc, La Jolla, CA, USA). If data were not normally distributed, non-parametric tests (Mann-Whitney *U* test) were used. A *p* value ≤ 0.05 was considered statistically significant.

## Results

### Characterization of the Ganglion Cell Layer in the PV-*Pten*^+/–^ Mouse Retina

**Figure 1:**
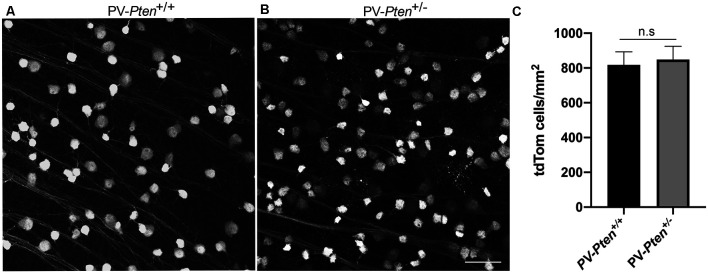
TdTomato fluorescence in the retina ganglion cell layer (GCL) of PV-*Pten* mice at 1.0 mm from the optic nerve head. TdTomato fluorescence was observed in cell bodies, dendrites, and axons with the strongest expression in the cell bodies. **(A)** TdTomato fluorescence in the GCL of a PV-*Pten*^ +/+^ mouse. **(B)** TdTomato fluorescence in the GCL of a PV-*Pten*^ +/–^ mouse. **(C)** Histogram indicating the mean (± SEM) density of tdTomato fluorescent cell bodies per mm^2^ in the GCL. z-step = 1 μm. Five optical sections were compressed for viewing. n.s., not significant. Scale bar: 50 μm.

In PV-*Pten*^+/+^ and PV-*Pten*^+/–^ retinas, tdTomato fluorescence was found in retinal neurons. tdTomato fluorescence (Figures 1A,B) was observed in the somata, dendrites, and axons, with the strongest expression in the soma. TdTomato fluorescence was localized to small-to large-diameter somata in the ganglion cell layer (GCL), ranging from 6–24 μm (*n* = 950 cells from 3 retinas). The density of tdTomato expressing somata in the PV-*Pten*^+/+^ (*n* = 3 retinas, 12 retinal regions), and PV-*Pten*^+/–^ (*n* = 4 retinas, 12 retinal regions) retinas was 818 ± 162 cells/mm^2^ and 849 ± 173 cells/mm^2^, respectively (Figure 1C, *P* > 0.05, Mann-Whitney test). These cell densities are consistent with the density of PV-immunoreactive ganglion cells in wild type mice (Kim and Jeon, 2006).

**Figure 2:**
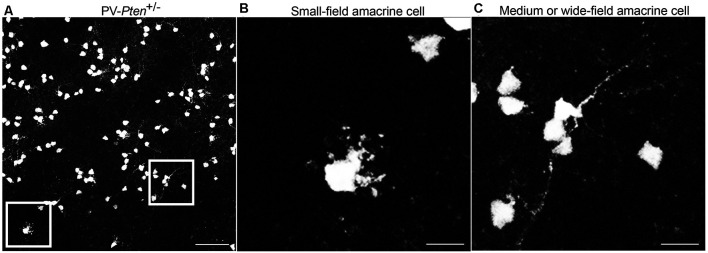
Expression of tdTomato fluorescence in the inner nuclear layer (INL) of PV-*Pten* mice. **(A)** TdTomato fluorescence in the INL of a PV-*Pten*^ +/–^ mouse. **(B)** High magnification (box area from **A**) of a small field amacrine cell. **(C)** High magnification (box area from **A**) of a medium or wide-field amacrine cell. *z*-step = 0.5 μm. Twenty optical sections were compressed for viewing. Scale bar: 50 μm.

TdTomato fluorescence was also observed in somata in the inner nuclear layer (INL) adjacent to the inner plexiform layer (IPL) in PV-*Pten*^+/+^ and PV-*Pten*^+/–^ retinas. Somal sizes ranged from 4.4–11.2 μm (*n* = 340 cells from three retinas). These neurons are likely amacrine cells due to their small somal diameter and location close to the INL/IPL border (Figure 2). TdTomato fluorescence was strong enough to visualize primary dendrites located in the IPL. The PV-*Pten* amacrine cell population is comprised of at least two different types (Figures 2A–C); a small-field amacrine cell with numerous varicosities (Figure 2B), and a second medium- or wide-field amacrine cell with longer and thin primary dendrites (Figure 2C). The morphological features of the small-field amacrine cell type are similar to previous descriptions of AII amacrine cells (Casini et al., 1995; Wässle et al., 1995, 2009; Massey and Mills, 1999; Pang et al., 2012).

**Figure 3:**
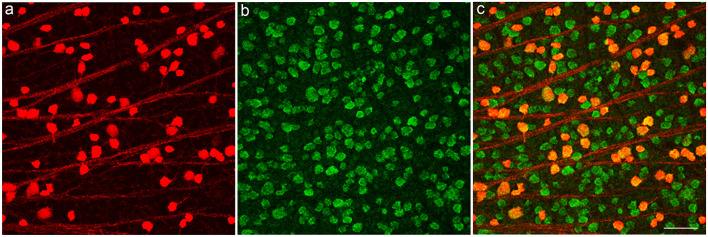
TdTomato fluorescent cells in GCL of a PV-*Pten*^ +/–^ mouse retina express the ganglion cell marker, RNA binding protein with multiple splicing (RBPMS). **(A)** TdTomato fluorescent ganglion cell bodies. **(B)** RBPMS-immunoreactive ganglion cell bodies in the GCL. **(C)** Merged images show that tdTomato fluorescent cell bodies contain RBPMS immunoreactivity. z-step = 1 μm. Three optical sections were compressed for viewing. Scale bar: 50 μm.

To characterize the tdTomato fluorescent cells in the GCL in the PV-*Pten* mouse line, whole mounts (Figures 3A–C) were immunostained with antibodies to retinal binding protein with multiple splicing (RBPMS), a pan-ganglion cell marker (Rodriguez et al., 2014). In these experiments, most tdTomato fluorescent cells expressed RBPMS, indicating that the majority (98% of cells, *n* = 3 retinas) are RGCs. In addition to the RGCs, 2% of the tdTomato fluorescent cells were not stained. They had smaller cell bodies, ranging from 6 to 10 μm with an average cell diameter of 8 ± 1 μm (*n* = 19 cells from three retinas). The small soma size and the lack of RBPMS immunostaining indicates the presence of a small number of PV-displaced amacrine cells that express tdTomato. This is consistent with a previous report that has also shown the presence of PV-positive displaced amacrine cells in the mouse retina (Kim and Jeon, 2006).

### Morphology and Tracer Coupling of α-RGCs

To analyze if a single-copy loss affects RGC morphology and tracer-coupling circuitry, we injected PV-tdTomato somata in the GCL with Neurobiotin and Lucifer Yellow. Neurobiotin was confirmed to have filled the cells when tapering dendritic endings were observed.

From the 40 or more morphological, transcriptional, and function subtypes of RGCs (Sanes and Masland, 2015; Baden et al., 2016; Tran et al., 2019), we focused on α-RGCs, a well-characterized ganglion cell type with large somata, dendritic stratifications in the ON and OFF layers of the IPL, and dendritic trees that form a circular to elliptical field (Sun et al., 2002; Völgyi et al., 2009; Sanes and Masland, 2015). In addition, the gap junction patterns of these cells are established in the mouse retina (Schubert et al., 2005; Völgyi et al., 2005). OFF α-RGCs are coupled to OFF α-RGCs in the GCL and to amacrine cells in the INL (Schubert et al., 2005; Völgyi et al., 2005). ON α-RGCs are only coupled to displaced amacrine cells and never exhibit coupling to other α-RGCs nor are they coupled to any cells in the INL (Schubert et al., 2005; Völgyi et al., 2005).

#### α-RGC Morphology

**Figure 4:**
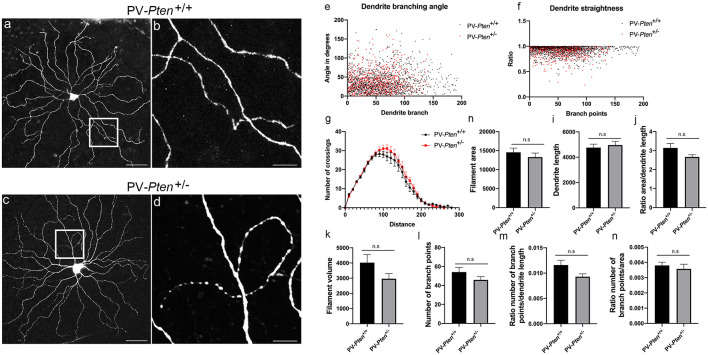
Morphology of ON α-RGCs in PV-*Pten*^+/+^ and PV-*Pten*^+/–^ retinas. **(A)** Neurobiotin-filled ON α-RGC in a PV-*Pten*^+/+^ retina showing the dendritic architecture. **(B)** High magnification (box area from **A**) of dendrites showing little overlap. **(C)** Neurobiotin-filled ON α-RGC in the PV-*Pten*^+/–^ retina showing the dendritic architecture. **(D)** High magnification (box area from **C**) of dendrites showing a loop-forming dendrite. Comparison of dendritic morphology of ON α-RGCs in PV-*Pten*^+/+^ (*n* = 14 cells) and PV-*Pten*^+/–^ (*n* = 11 cells) retinas. **(E)** Dendrite branching angle (*P* > 0.05, Mann-Whitney test). **(F)** Dendrite straightness (*P* > 0.05, Mann-Whitney test). **(G)** Sholl analysis (*P* > 0.05 at all radii, Mann-Whitney test). **(H)** Filament area (*P* > 0.05, Mann-Whitney test). **(I)** Dendrite length (*P* > 0.05, Mann-Whitney test). **(J)** Ratio area to dendrite length (*P* > 0.05, Mann-Whitney test). **(K)** Volume (*P* > 0.05, Mann-Whitney test). **(L)** Branch points (*P* > 0.05, Mann-Whitney test). **(M)** Ratio of branch points to dendrite length (*P* > 0.05, Mann-Whitney test). **(N)** Ratio to branch points to area (*P* > 0.05, Mann-Whitney test). z-step = 0.3 μm. 40–60 optical sections were compressed for viewing. Scale bars: 50 μm **(A,C)**, 10 μm **(B,D)**. n.s., not significant.

ON α-RGC dendrites in the PV-*Pten*^+/+^ mice (*n* = 14 cells, Figures 4A,B) stratified in sublamina b of the IPL, close to the GCL border and formed a circular field. We observed dendrites turning back towards the cell body to form loops in the majority of PV-*Pten*^+/–^ mice (*n* = 7/11 cells, Figures 4C,D). In contrast, in PV-*Pten*^+/+^ retinas, loop-forming dendrites were observed in two out of 14 ON α-RGCs. Morphological analysis of the Neurobiotin labeled ON α-RGCs was performed using the Imaris 9.5.0 software (Bitplane AG, Concord, MA, USA), which provided information on filaments-dendrite branching angle B (Figure 4E), filament-dendrite straightness (Figure 4F), Sholl analysis (Figure 4G), filament area (Figure 4H), dendrite length (Figure 4I), ratio area/dendrite length (Figure 4J), filament volume (Figure 4K), number of dendrite branch points (Figure 4L), ratio number of branch points/dendrite length (Figure 4M), and ratio number of branch points/area (Figure 4N, see “Materials and Methods” section). ON α-RGCs in the PV-*Pten*^+/–^ mice did not show significant differences compared to ON α-RGCs in the PV-*Pten*^+/+^ mice in any of these morphological parameters (Figures 4E–N, *P* > 0.05, Mann-Whitney test).

**Figure 5:**
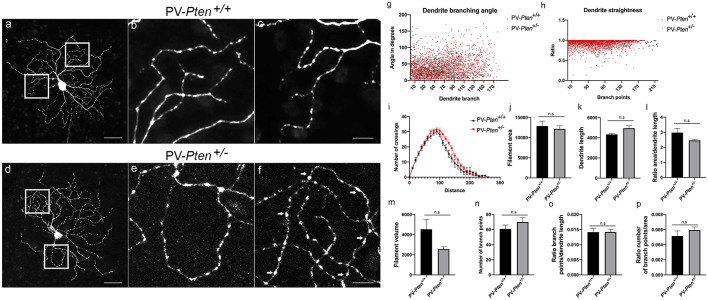
Morphology of OFF α-RGCs in PV-*Pten*^+/+^ and PV-*Pten*^+/–^ retinas. **(A)** Neurobiotin-filled OFF α-RGC in a PV-*Pten*^+/+^ retina showing the dendritic architecture. **(B)** High magnification (box area from **A**) of dendrites showing little overlap. **(C)** Another high magnification example (box area from **A**) of dendrites showing little overlap. **(D)** Neurobiotin-filled OFF α-RGC in the PV-*Pten*^+/–^ retina showing dendritic architecture. **(E)** High magnification (box area from **C**) of dendrites showing a loop-forming dendrite. **(F)** Another high magnification example (box area from **A**) of dendrites showing a loop-forming dendrite (arrows). Comparison of dendritic morphology of OFF α-RGCs in PV-*Pten*^+/+^ (*n* = 9 cells) and PV-*Pten*^+/–^ (*n* = 12 cells) retinas. **(G)** Dendrite branching angle (*P* > 0.05, Mann-Whitney test). **(H)** Dendrite straightness (*P* > 0.05, Mann-Whitney test). **(I)** Sholl analysis (*P* > 0.05 at all radii, Mann-Whitney test). **(J)** Filament area (*P* > 0.05, Mann-Whitney test). **(K)** Dendrite length (*P* > 0.05, Mann-Whitney test). **(L)** Ratio area to dendrite length (*P* > 0.05, Mann-Whitney test). **(M)** Volume (*P* > 0.05, Mann-Whitney test). **(N)** Branch points (*P* > 0.05, Mann-Whitney test). **(O)** Ratio of branch points to dendrite length (*P* > 0.05, Mann-Whitney test). **(P)** Ratio to branch points to area (*P* > 0.05, Mann-Whitney test). z-step = 0.3 μm. 80–95 optical sections were compressed for viewing. Scale bars: 50 μm **(A,D)**, 10 μm **(B,C,E,F)**. n.s., not significant.

OFF α-RGC dendrites in the PV-*Pten*^+/+^ mice (*n* = 10 cells, Figures 5A–C) usually had an elliptical dendritic field, although some RGCs with a circular field were also observed. The dendrites rarely overlapped in PV-*Pten*^+/+^ retinas (Figures 5B,C). Loop-forming dendrites were observed in about a third of the PV-*Pten*^+/+^ OFF α-RGCs (*n* = 3/10 cells). In contrast, more than half of the injected cells in the PV-*Pten*^+/–^ mice exhibited loop-forming dendrites (*n* = 14/23 cells, Figures 5D–F arrows), similar to the ON α-RGCs. Consistent with the lack of morphological differences in the ON α-RGCs, OFF α-RGCs in the PV-*Pten*^+/–^ mice also did not show significant differences in their morphology compared to OFF α-RGCs in the PV-*Pten*^+/+^ mice (Figures 5G–P, *P* > 0.05, Mann-Whitney test).

#### α-RGCs Coupling Patterns

**Figure 6:**
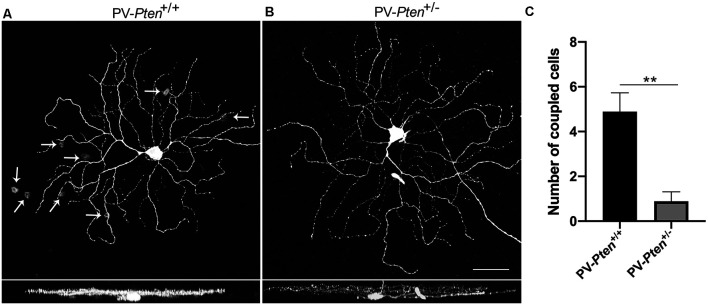
Tracer coupling of ON α-RGCs in PV-*Pten*^+/+^ and PV-*Pten*^+/–^ retinas. **(A)** Neurobiotin-filled ON α-RGC in PV-*Pten*^+/+^ retina and its stratification pattern. The ON α-RGC shows tracer-coupling to small cells in the GCL (arrows). **(B)** Neurobiotin-filled ON α-RGC in a PV-*Pten*^+/–^ retina and its stratification pattern. The ON α-RGC shows no tracer-coupling. **(C)** Histogram indicating the mean (± SEM) number of cells coupled to ON α-RGCs (*n* = 18 cells from PV-*Pten*^+/+^ mice, *n* = 9 cells from PV-*Pten*^+/–^ mice, *P* < 0.005, Mann-Whitney test). z-step = 0.3 μm. 40–60 optical sections were compressed for viewing. Scale bar = 50 μm. **P < 0.005.

ON α-RGCs in the PV-*Pten*^+/+^ retinas were coupled to displaced amacrine cells (*n* = 18 cells, Figure 6A). We observed 4.9 ± 0.8 coupled displaced amacrine cells, with an average somal diameter of 9.4 ± 0.6 μm (*n* = 41 cells). In contrast, ON α-RGCs in the PV-*Pten*^+/–^ retinas were either coupled to a displaced amacrine cell in the GCL (*n* = 4 cells) or uncoupled (*n* = 5 cells, Figure 6B). The average number of coupled amacrine cells in the GCL was 2 ± 0.6 cells (*n* = 4 cells), with an average somal diameter of 10 ± 1 μm (*n* = 8 cells). The decrease in cell coupling in the GCL was significant (*P* < 0.005; Mann-Whitney test, Figure 6C).

**Figure 7:**
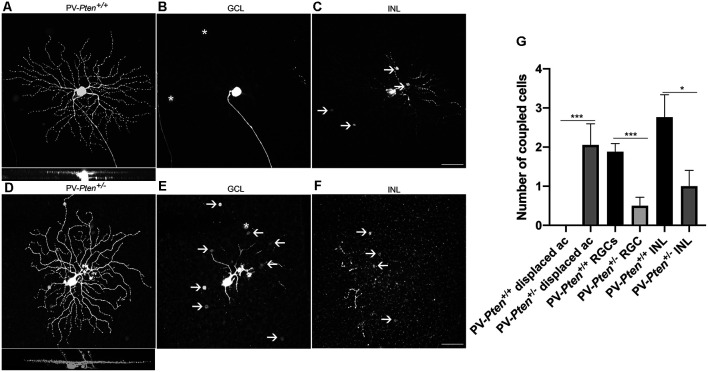
Tracer coupling of OFF α-RGCs in PV-*Pten*^+/+^ and PV-*Pten*^+/–^ retinas. **(A)** Neurobiotin-filled OFF α-RGC in PV-*Pten*^+/+^ retina and its stratification pattern. **(B)** OFF α-RGC shows tracer-coupling to neighboring RGCs in the GCL (asterisk) in PV-*Pten*^+/+^ retina. **(C)** OFF α-RGC shows tracer-coupling to amacrine cells in the INL (arrows) in PV-*Pten*^+/+^ retina. **(D)** Neurobiotin-filled OFF α-RGC in a PV-*Pten*^+/–^ retina and its stratification pattern. Asterisks show mis-injected Müller cells. **(E)** OFF α-RGCs show tracer-coupling to ganglion cells (asterisks) and displaced amacrine cells (arrows) in the GCL in PV-*Pten*^+/–^ retina. **(F)** OFF α-RGC shows tracer-coupling to amacrine cells in the INL (arrows) in PV-*Pten*^+/–^ retina. **(G)** Histogram indicating the mean (± SEM) number of cells coupled to OFF α-RGCs (*n* = 17 cells from PV-*Pten*^+/+^ mice, *n* = 18 cells from PV-*Pten*^+/–^ mice, *P < 0.05, ***P < 0.001, Mann-Whitney test). z-step = 0.3 μm. 75–90 optical sections were compressed for viewing. Scale bar = 50 μm.

OFF α-RGCs in the PV-*Pten*^+/+^ retinas were coupled to other OFF α-RGCs in the GCL (*n* = 17 cells, Figures 7A,B) and to amacrine cells in the INL (Figures 7A,C), as reported previously (Schubert et al., 2005; Völgyi et al., 2005). The number of coupled RGCs to OFF α-RGCs in the GCL was 1.9 ± 0.2 cells, with an average of somal diameter of 16.3 ± 0.4 μm (*n* = 19 cells) in PV-*Pten*^ +/+^ retinas. The number of coupled amacrine cells in the INL was 2.8 ± 0.6 cells, with an average somal diameter of 7.7 ± 0.3 μm (*n* = 28 cells). In contrast, OFF α-RGCs in the PV-*Pten*^+/–^ retinas (*n* = 18 cells) were coupled to either two different cell types, or a single cell type based on their somal size in the GCL (Figures 7D,F). One cell type was the OFF α-RGC. The other cells were displaced amacrine cells. There was a significant reduction in cell coupling to other OFF α-RGCs (*P* < 0.0001; Mann-Whitney test, Figures 7E,G) compared to OFF α-RGCs in PV-*Pten*^+/+^ retinas. The average number of coupled RGCs to OFF α-RGCs was 0.5 ± 0.2 cells in the GCL and their average somal diameter was 16.8 ± 0.9 μm (*n* = 4 cells). The average number of coupled cells to displaced amacrine cells (*P* < 0.0001; Mann-Whitney test, Figures 7E,G) was 2.1 ± 0.5 cells in the GCL with an average somal diameter of 8.5 ± 0.3 μm (*n* = 12 cells). There was also a significant reduction in cell coupling to amacrine cells in the INL compared to PV-*Pten*^+/+^ retinas (*P* < 0.05; Mann-Whitney test, Figures 7F,G). The average number coupled amacrine cells in the INL was 1 ± 0.4 cells with an average somal diameter of 7.3 ± 0.4 μm (*n* = 12 cells).

### Cx36 Expression in OFF α-RGCs

Next, we examined gap junction connexin (Cx) expression in OFF α-RGCs. Cx36 is the most abundant Cx in the retina and mediates coupling among the majority of RGC types, including OFF α-RGCs (Schubert et al., 2005; Völgyi et al., 2005; Pan et al., 2010).

**Figure 8:**
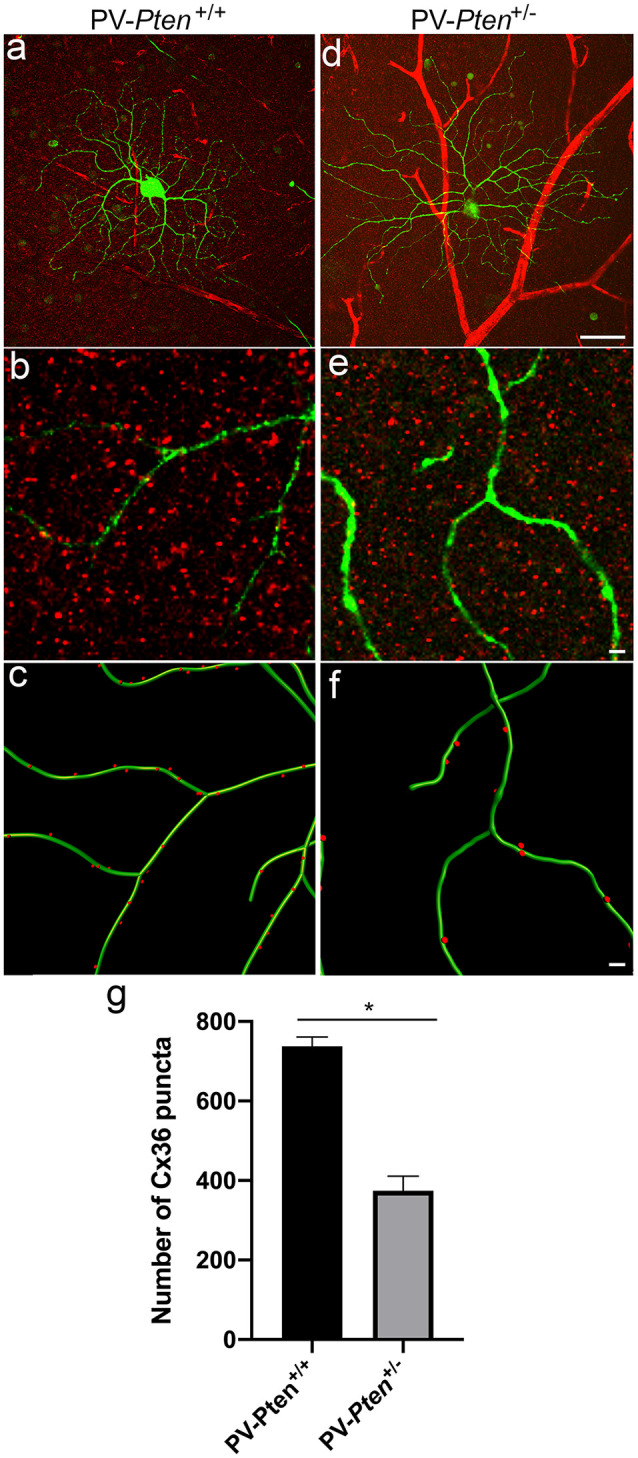
Cx36 expression in OFF α-RGCs in PV-*Pten*^+/+^ and PV- *Pten*^+/–^ retinas. **(A)** A maximum projection of a Neurobiotin-filled OFF α-RGC (green) stained with Cx36 antibodies (red) in a PV-*Pten*^+/+^ retina at 1.0 mm from the optic nerve head. **(B)** A single optical section of the Neurobiotin-filled OFF α-RGC (green) stained with Cx36 antibodies (red) in PV-*Pten*^+/+^ retina. **(C)** Imaris reconstruction of panel **(B)**. Cx36 puncta closer than 0.3 μm to the dendrites are represented in red. **(D)** A maximum projection of a Neurobiotin-filled OFF α-RGC (green) stained with Cx36 antibodies (red) in PV-*Pten*^+/–^ retina at 1.0 mm from the optic nerve head. **(E)** A single optical section of the Neurobiotin-filled OFF α-RGC (green) stained with Cx36 antibodies (red) in PV-*Pten*^+/–^ retina. **(F)** Imaris reconstruction of panel **(E)**. Cx36 puncta closer than 0.3 μm to the dendrites are represented in red. **(G)** Histogram indicating the mean (± SEM) of Cx36 puncta in OFF α-RGCs (*n* = 3 cells from PV-*Pten*^ +/+^ retinas, *n* = 5 cells from PV-*Pten*^+/–^ retinas, *P < 0.05, Mann-Whitney test). Scale bar in **(A,D)** = 50 μm. Scale bar in **(B,C,E,F)** = 2 μm.

To determine the density of gap junctions on ganglion cell dendrites, the distribution of Cx36 puncta was analyzed in injected OFF α-RGCs located in middle regions of the retina (*n* = 3 cells from three PV-*Pten*^+/+^ mice; *n* = 5 cells from three PV-*Pten*^+/–^ mice) stained with a specific antibody against Cx36 (Figure 8). Overall, the OFF α-RGCs had an average of 737.3 ± 24 Cx36 puncta (Figures 8A,C,E) compared to 374.2 ± 37 Cx36 puncta in OFF α-RGCs in the PV-*Pten*^+/–^ mice (Figures 8B,D,E). This corresponded to a 50% reduction of Cx36 puncta in OFF α-RGC in the PV-*Pten*^+/–^ mice (*P* < 0.05, Mann-Whitney test).

**Figure 9:**
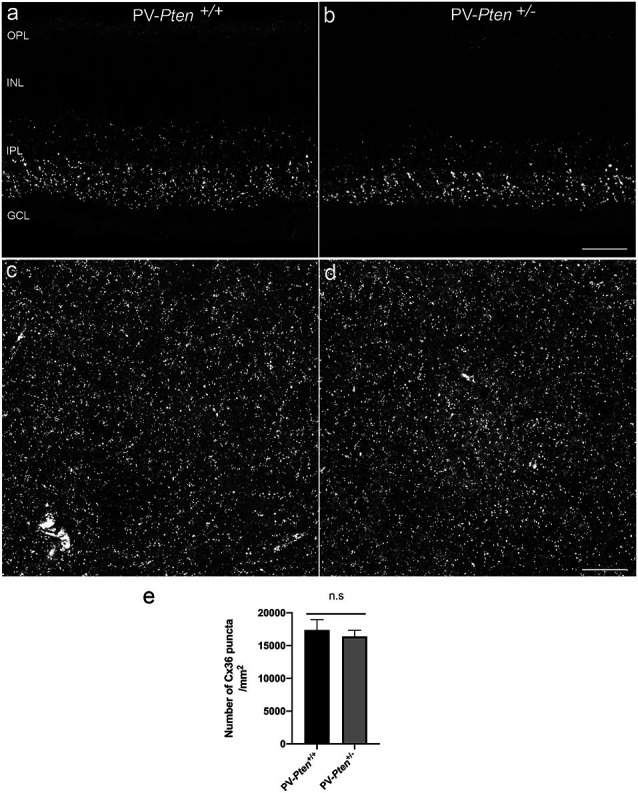
Cx36 immunoreactivity in PV-*Pten*^+/+^ and PV-*Pten*^+/–^ retinas. **(A)** Cx36 immunoreactivity in retinal sections in PV-*Pten*^+/+^. **(B)** Cx36 immunoreactivity in retinal sections in PV-*Pten*^ +/–^. **(C)** A maximum projection of Cx36 immunoreactivity in PV-*Pten*^ +/+^ wholemount retinas at 1.0 mm from the optic nerve head. **(D)** A maximum projection of Cx36 immunoreactivity in PV-*Pten*^+/–^ wholemount retinas at 1.0 mm from the optic nerve head. **(E)** Histogram indicating the mean (± SEM) of Cx36 puncta in PV-*Pten*^+/+^ and PV-*Pten*^ +/–^ whole mounted retinas (*n* = 9 PV-*Pten*^+/+^ retinas; *n* = 3 PV-*Pten*^+/–^ retinas, *P* > 0.05, Mann-Whitney test). z-step = 0.3 μm. Four **(A,B)** and 16 **(C,D)** optical sections were compressed for viewing. n.s., not significant. Scale bar = 50 μm.

We also used a Cx36 antibody to investigate the overall pattern of Cx36 puncta in the IPL of the PV-*Pten* mouse retinas. In vertical sections (Figures 9A,B), there was weak Cx36 immunoreactivity in the outer plexiform layer (OPL), and small immunoreactive puncta were observed in the IPL. The brightest immunostained puncta were found in the ON sublamina of the IPL of the PV-*Pten*^+/+^ (Figure 9A; *n* = 3 retinas) and PV-*Pten*^+/–^ retinas (Figure 9B; *n* = 3 retinas), consistent with previous studies (Güldenagel et al., 2000, 2001; Feigenspan et al., 2001; Mills et al., 2001; Deans et al., 2002).

Semi-quantification of the Cx36 expression in the IPL were done in both PV-*Pten*^+/+^ (Figure 9C; *n* = 9 retinas) and PV-*Pten*^+/–^ (Figure 9D; *n* = 3 retinas) whole-mounted retinas. The IPL of PV-*Pten*^+/+^ retinas contained 16402 ± 917/mm^2^ Cx36 puncta while the PV-*Pten*^+/–^ contained 17370 ± 1590/mm^2^ (Figure 9E). Although the overall number of Cx36 puncta was lower in the PV-*Pten*^+/–^ mice compared to PV-*Pten*^+/+^ mice, these differences were not significant (*P* > 0.05).

### Amacrine and Bipolar Cell Populations in the PV-*Pten* Mouse Line

*Pten* is a positive regulator of amacrine cell genesis (Tachibana et al., 2016), and suppression of PI3K/Akt signaling by *Pten* is crucial for proper neuronal differentiation and forming normal neuronal networks (Sakagami et al., 2012; Tachibana et al., 2016). Since PV-amacrine cells are expressed in the PV-*Pten*^+/–^ retinas, we studied well-characterized amacrine and bipolar cell populations in the INL to determine if amacrine cell production is affected when a copy of *Pten* is deleted.

*Amacrine cell populations*: Vertical sections were immunostained with specific antibodies directed against choline acetyltransferase (ChAT) to identify cholinergic amacrine cells (Kang et al., 2004), Prox1 to identify AII amacrine cells (Pérez de Sevilla Müller et al., 2010a; Keeley and Reese, 2018), glutamic acid decarboxylase (GAD67) to identify GABAergic amacrine cells (Schnitzer and Rusoff, 1984), glycine to identify glycinergic amacrine cells (Pourcho and Goebel, 1985), and calretinin to label amacrine, displaced amacrine and ganglion cells, including PV-cells (Haverkamp and Wässle, 2000; Haverkamp et al., 2009). No significant differences were observed in the number of GAD67, Prox1, ChAT, and calretinin positive cells in PV-*Pten*^+/–^ mice compared to PV-*Pten*^+/+^ mice (Table 2, Supplementary Figures 1A–L, *P* > 0.05, Mann-Whitney test). Glycine antibodies in whole-mounted retinas strongly labeled glycinergic amacrine cells in the INL and weakly labeled bipolar cells (Supplementary Figure 2), consistent with earlier findings (Menger et al., 1998; Vaney et al., 1998). The average density of glycinergic-expressing amacrine cells was 4125.6 ± 473.2/mm^2^ in PV-*Pten*^+/+^ (*n* = 4 retinas) compared to 4695 ± 1220 cells/mm^2^ in the PV-*Pten*^+/–^ mice (*n* = 3 retinas). No significant difference was found in the density of glycinergic amacrine cells (Supplementary Figure 2E; *P* > 0.05, Mann-Whitney test).

**Table 2:** Amacrine cell populations in the PV-*Pten* mouse line.

Amacrine cell marker	Density in PV-*Pten*^+/+^/500 μm	Density in PV-*Pten*^+/^^−^/500 μm	Mann-Whitney Test
GAD67	**INL**: 55.1 ± 12 cells **GCL**: 14.7 ± 3.4 cells *n* = 2 retinas	**INL**: 76 ± 5.8 cells **GCL**: 31.4 ± 5.2 cells *n* = 4 retinas	*P* > 0.05
Prox1	**INL**: 43.3 ± 8 cells *n* = 2 retinas	**INL**: 37.2 ± 5 cells *n* = 4 retinas	*P* > 0.05
ChAT	**INL**: 12.8 ± 1.9 cells **GCL**: 13.7 ± 1.3 cells *n* = 4 retinas	**INL**: 12 ± 2.3 cells **GCL**: 11.8 ± 1.6 cells *n* = 4 retinas	*P* > 0.05
Calretinin	**INL**: 45.2 ± 4.8 cells **GCL**: 26.7 ± 3.6 cells *n* = 3 retinas	**INL**: 43.2 ± 5.5 cells **GCL**: 30.2 ± 2.2 cells *n* = 2 retinas	*P* > 0.05

*Bipolar cell populations*: To study the bipolar cell population, we performed labeling experiments for Goα, a marker for ON-cone bipolar cells, PKCα, a marker for rod bipolar cells and the vesicular glutamate transporter 1 (VGluT-1), a marker for synaptic terminals of all bipolar cells as well as rod spherules and cone pedicles (Haverkamp and Wässle, 2000; Haverkamp et al., 2003; Johnson et al., 2003). We found no significant differences in the number of Goα-expressing bipolar cells and PKCα-expressing bipolar cells in PV-*Pten*^+/–^ mice compared to PV-*Pten*^+/+^ mice (Table 3; Supplementary Figures 3A–F; *P* > 0.05, Mann-Whitney test).

**Table 3:** Bipolar cell populations in the PV-*Pten* mouse line.

Bipolar cell marker	Density in PV-*Pten*^+/+^/500 μm	Density in PV- *Pten*^+/^^−^/500 μm	Mann-Whitney Test
Goα	**INL**: 86.7 ± 5.6 cells *n* = 2 retinas	**INL**: 90.5 ± 2.3 cells *n* = 2 retinas	*p* > 0.05
PKCα	**INL**: 84.7 ± 5.5 cells *n* = 2 retinas	**INL**: 79.6 ± 1.5 cells *n* = 2 retinas	*p* > 0.05

Immunostaining studies with the antibodies to VGluT-1 showed no differences in the immunostaining levels and intensity in the IPL and OPL of the PV-*Pten*^+/–^ retina (Supplementary Figures 3G,H; *n* = 2 retinas) compared to the PV-*Pten*^+/+^ retina.

In summary, these findings indicate that the lack of one *Pten* copy in PV-RGCs and PV-amacrine cells does not appear to alter the number of cells in several representative bipolar and amacrine cell types, or the pattern of photoreceptor and bipolar cell terminals in the plexiform layers.

## Discussion

Manipulation of the Pten pathway provides insight into its potential therapeutic use in eye diseases. Pten signaling promotes RGC axon regeneration and enhances RGC survival following ocular injury (Leibinger et al., 2012; de Lima et al., 2012; Duan et al., 2015); however, to date, there is no systematic study evaluating RGC morphology, connectivity, gap junction expression, and the impact on RGC connectivity in *Pten* deletion lines.

The present study examines the effects of a single-copy loss of *Pten* in specific PV retinal types, with a focus on α-RGCs. Suppression of PI3K/Akt signaling by Pten is crucial for proper retinal neuronal differentiation and normal circuitry formation (Sakagami et al., 2012; Tachibana et al., 2016), consistent with other studies that report cortical dendritic and synaptic changes with *Pten* deletion (Kwon et al., 2006; Chow et al., 2009; Xiong et al., 2012). Although α-RGCs did not exhibit changes in somal size and showed modest changes in dendritic morphology, the α-RGCs did show a significant decrease in the number of Cx36 immunoreactive puncta and a reduction in cell coupling compared to α-RGCs in littermate, PV-*Pten*^+/+^ retinas. The numerical reduction of RGC tracer coupling is likely due to the reduced expression of Cx36 in OFF α-RGCs. In addition, most OFF α-RGCs showed altered changes in their connectivity, with aberrant gap junction connectivity to displaced amacrine cells. This altered connectivity (Figures 7E,G) is likely to result in changes in α-RGC functional properties that would impact on visual image processing.

### Reduced Coupling in PV-RGCs

Preiss et al. (2007) suggested that classical PKC isoforms may be involved in signaling to Akt phosphorylation. In addition, protein kinases are responsible for the phosphorylation of the connexins (Xia and Mills, 2004; Urschel et al., 2006; Pérez de Sevilla Müller et al., 2010a). Therefore, we hypothesize that *Pten* signaling modulates gap junction coupling by affecting protein kinase phosphorylation. Moreover, confocal microscopy and immunoprecipitation assays have shown that Cx43 binds to PTEN (González-Sánchez et al., 2016) and that the antiproliferative effect of Cx43, the major protein forming gap junctions in astrocytes, is reduced in glioma cells and astrocytes when *Pten* levels are reduced using NT-siRNA approaches (González-Sánchez et al., 2016). These experimental findings are consistent with our observations that Cx36 neuronal expression and gap junctional connectivity measured by tracer coupling is also affected by reduction of *Pten* signaling in retinal cells with a single copy of *Pten*.

In the mouse retina, Cx36 mediates coupling of the majority of RGCs (Pan et al., 2010), which underlies the synchronization of activity of neighboring RGCs. Homologous coupling between RGC neighbors is believed to underlie short-latency synchrony of impulse activity, whereas the heterologous coupling between RGCs and amacrine cells results in broader and correlated activity (Mastronarde, 1983a,b,c; Meister et al., 1995; Brivanlou et al., 1998; DeVries, 1999; Meister and Berry, 1999; Hu and Bloomfield, 2003; Völgyi et al., 2013b). A reduction in coupling due to the lack of one *Pten* gene could impact intracellular communication and have a deleterious influence on visual information processing. For instance, the loss of spike correlations and synchrony from gap junctions will likely decrease the propagation of visual signals (Alonso et al., 1996; Stevens and Zador, 1998; Singer, 1999; Usrey and Reid, 1999) as well as the short-latency spike synchrony in RGCs (Arnett and Spraker, 1981; Brivanlou et al., 1998; DeVries, 1999; Hu and Bloomfield, 2003).

Although OFF α-RGCs showed a decrease in the number of Cx36 immunoreactive puncta, the overall pattern of Cx36 puncta in the IPL remained the same. A possible explanation could be the formation of the aberrant Cx36 gap junctions with displaced amacrine cells, since their formation between OFF α-RGCs with a single-copy loss of *Pten* and displaced amacrine cells requires these amacrine cells to express Cx36. An increase in Cx gene expression has also been demonstrated in a model of neuroinflammation in the rat hippocampus (Abbasian et al., 2012). Additionally, Cx43 is upregulated following central nervous system injury (Danesh-Meyer et al., 2012). Based on these other pathological observations, changes in PTEN signaling in OFF α-RGCs lacking a single copy of *Pten* could also impact on the regulation of Cx36 gene expression in these ganglion cells as well as altering connectivity and influencing Cx36 expression in other retinal cell types. In addition to the altered cellular connectivity of the α-RGCs, the connectivity of other RGCs is also likely to be changed with reduction of PTEN gene expression. The altered connectivity and presumably altered functional properties as shown in cortical pyramidal neurons (Garcia-Junco-Clemente et al., 2013) would potentially impact the efficacy of future therapeutic approaches that manipulate the *Pten* signaling pathway for treating ophthalmic diseases.

It is important to note that four αRGC types have been described in the mouse retina based on responses to light steps: ON-sustained, ON-transient, OFF-sustained, and OFF-transient (Pang et al., 2003; Van Wyk et al., 2009; Krieger et al., 2017; Sawant et al., 2021). Morphologically, they differ in the level of dendritic stratification within the IPL with the ON types that ramify closer to the GCL and the OFF types closer to the INL (Pang et al., 2003; Van Wyk et al., 2009; Krieger et al., 2017; Sawant et al., 2021). The variety of α-RGC types could explain some of the differences we observed in the PV-*Pten*^+/–^ retinas. Twelve OFF α-RGCs formed novel coupling to displaced amacrine cells while six other OFF α-RGCs were either uncoupled or with a significant reduction of their normal coupling patterns. The fact that some OFF α-RGCs had an elliptical dendritic field, or a circular field could be also due to different types of OFF α-RGCs.

While our data for the ON α-RGCs is quite consistent, another aspect to consider is that ON-sustained α-RGCs display a nasal-to-temporal gradient in cell density, size, and receptive fields (Bleckert et al., 2014). These changes in the retina might also impact in their gap junction patterns and the overall number of Cx36 expression in ON α-RGCs depending on the nasal-to-temporal gradient.

In addition to alterations of gap junction connectivity of the PV-positive neurons in the PV-*Pten*^+/–^ retinas, other signaling and cellular changes might occur in PV-positive neurons. *Pten* haploinsufficiency in cortical pyramidal neurons increases the expression of small conductance calcium-activated potassium (SK) channels, resulting in an increase in the amplitude of the after-spike hyperpolarization and a decrease in intrinsic excitability (Garcia-Junco-Clemente et al., 2013). The change in intrinsic excitability reduces the evoked firing rates of cortical pyramidal neurons (Garcia-Junco-Clemente et al., 2013). With many known calcium-activated potassium channels expressed in RGCs (Wang et al., 1998) and amacrine cells (Grimes et al., 2009; Tanimoto et al., 2012), we speculate that PV-positive retinal neurons in the PV-*Pten*^+/–^ mice could also have this channelopathy and a decrease in RGC intrinsic excitability. Furthermore, calcium-activated potassium channels often co-localize with Ca^2+^ channels to regulate Ca^2+^ levels (Lee and Cui, 2010; Van Hook et al., 2019), suggesting the possibility that this channelopathy might also affect Ca^2+^ channel function, and alter both intrinsic and extrinsic cellular signaling.

## Conclusions

Precise electrical and chemical synaptic organization between retinal neurons is important for proper neural network function and visual transmission to the brain (Varadarajan and Huberman, 2018). Changes in neural wiring from disease or trauma are thus likely to alter visual information processing (Strettoi and Pignatelli, 2000; Cuenca et al., 2005; Gargini et al., 2007; Puthussery et al., 2009; Phillips et al., 2010). Altered gap junctional connectivity in the inner retina, together with functional changes in cortical cell responsivity reported for pyramidal neurons with one copy of *Pten* deleted (Garcia-Junco-Clemente et al., 2013; Baohan et al., 2016) presents a potential barrier for implementing *Pten*-related therapeutic interventions in eye diseases. These findings suggest caution in evaluating the therapeutic potential of findings that manipulation of the PTEN pathway to enhance RGC survival and promote axon regeneration (Park et al., 2008; Sun et al., 2011; de Lima et al., 2012; Duan et al., 2015; Norsworthy et al., 2017; Li et al., 2018; Wang et al., 2018). A possible approach for manipulation of the PTEN pathway would be to identify possible windows of intervention during early stages of retinal remodeling (Jones and Marc, 2005; Cuenca et al., 2014) and careful implementation of therapeutic protocols to modulate the PTEN pathway to prevent or treat visual-related abnormalities in neurodegenerative diseases.

## Data Availability Statement

The original contributions presented in the study are included in the article/Supplementary Material, further inquiries can be directed to the corresponding author.

## Ethics Statement

The animal study was reviewed and approved and these studies were conducted under protocols approved by the University of California at Los Angeles (UCLA) Animal Research Committee. All experiments were carried out in accordance with guidelines for the welfare of experimental animals issued by the U.S. Public Health Service Policy on Human Care and Use of Laboratory Animals, and the UCLA Animal Research Committee.

## Author Contributions

LPS conceived the project, designed the experiments, and supervised the project. LPS, AC, and SA performed the experiments. LPS, AC, SA, and AH analyzed the data. LPS, SA, and NB wrote the article. All authors contributed to the article and approved the submitted version.

## Conflict of Interest

The authors declare that the research was conducted in the absence of any commercial or financial relationships that could be construed as a potential conflict of interest.
