# Supplementary material for: PTEN Expression Regulates Gap Junction Connectivity in the Retina
Source: Front Neuroanat. 2021 May 20;15:629244. doi: 10.3389/fnana.2021.629244 (PMC8172595; doi:10.3389/fnana.2021.629244)
Supplement: Supplementary file 1 [file Data_Sheet_1.PDF]

Supplementary Material

1 Supplementary Data

Supplementary Figures

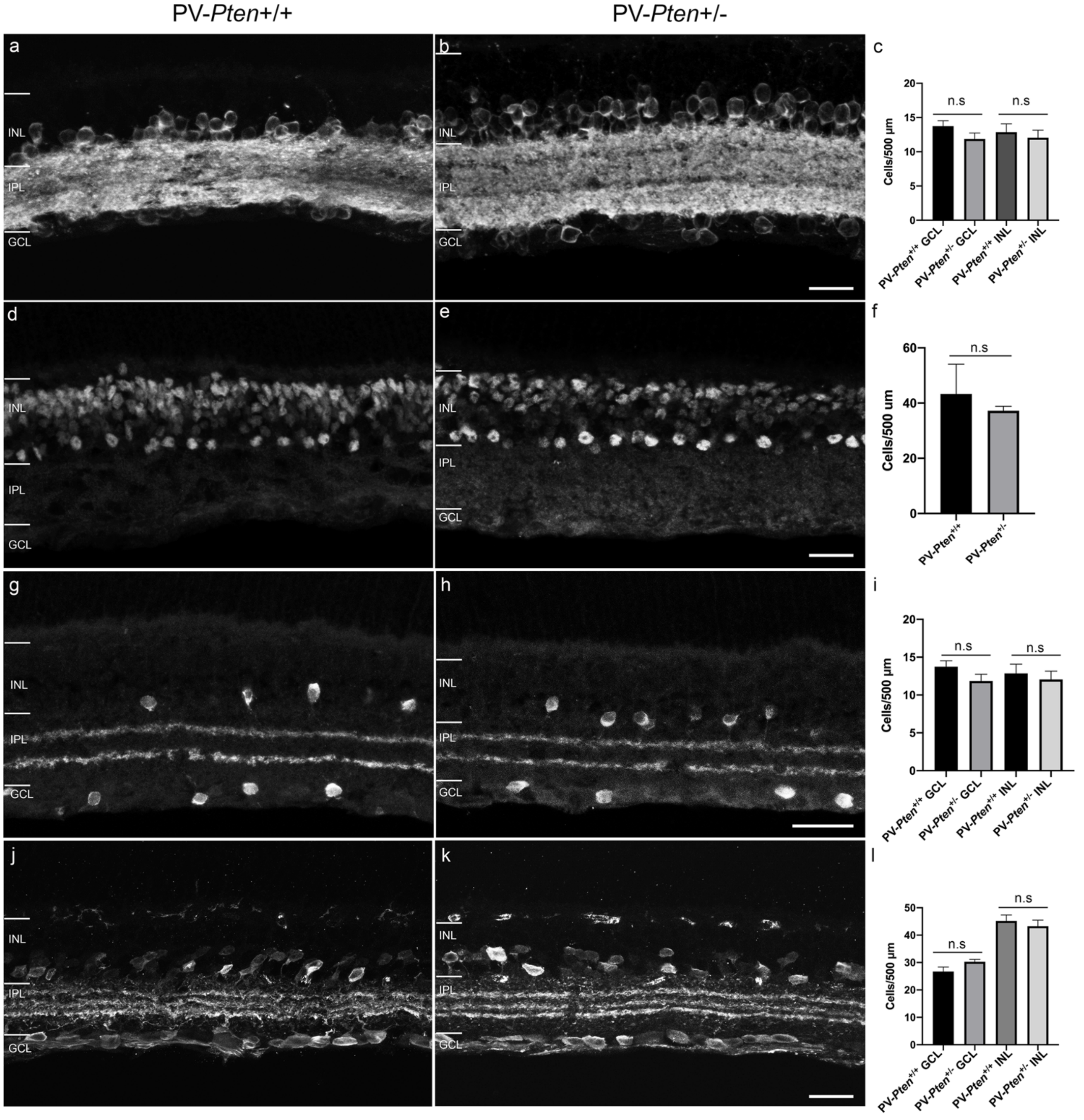

Supplementary Figure 1. GAD67, Prox1, ChAT and calretinin immunoreactivity in PV-*Pten*$^{+/+}$ and PV-*Pten*$^{+/-}$ retinal sections. (a) GAD67 immunoreactivity in cell bodies in the INL and GCL in PV-*Pten*$^{+/+}$ retinas. (b) GAD67 immunoreactivity in cell bodies in the INL and GCL in PV-*Pten*$^{+/-}$ retinas. (c) Histogram indicating the mean (± SEM) of GAD67 positive cells per 500 µm in PV-*Pten*$^{+/+}$ (n=2 retinas) and PV-*Pten*$^{+/-}$ retinas (n= 4 retinas, $P > 0.05$, Mann-Whitney test). (d) Prox1 immunoreactivity in amacrine and bipolar cell bodies in PV-*Pten*$^{+/+}$ retinas. (e) Prox1 immunoreactivity in amacrine and bipolar cell bodies in PV-*Pten*$^{+/-}$ retinas. (f) Histogram indicating the mean (± SEM) of Prox1 positive cells per 500 µm in PV-*Pten*$^{+/+}$ (n= 2 retinas) and PV-*Pten*$^{+/-}$

retinas (n= 4 retinas, P > 0.05, Mann-Whitney test). (g) ChAT immunoreactivity in amacrine cell bodies in PV-*Pten* $^{+/+}$ retinas. (h) ChAT immunoreactivity in amacrine cell bodies in PV-*Pten*$^{+/–}$ retinas. (i) Histogram indicating the mean (± SEM) of ChAT positive cells per 500 µm in PV-*Pten*$^{+/+}$ (n=4 retinas) and PV-*Pten*$^{+/–}$ retinas (n= 4 retinas, P > 0.05, Mann-Whitney test). (j) Calretinin immunoreactivity in cell bodies in the GCL and INL in PV-*Pten*$^{+/+}$ retinas. (k) Calretinin immunoreactivity in cell bodies in the GCL and INL in PV-*Pten*$^{+/–}$ retinas. (l) Histogram indicating the mean (± SEM) of calretinin positive cells per 500 µm in PV-*Pten*$^{+/+}$ (n=3 retinas) and PV-*Pten*$^{+/–}$ retinas (n= 2 retinas, P > 0.05, Mann-Whitney test). z-step = 1 µm; 3–5 optical sections were compressed for viewing. INL, Inner nuclear layer; IPL, Inner plexiform layer; GCL, Ganglion cell layer. Scale bar = 50 µm.

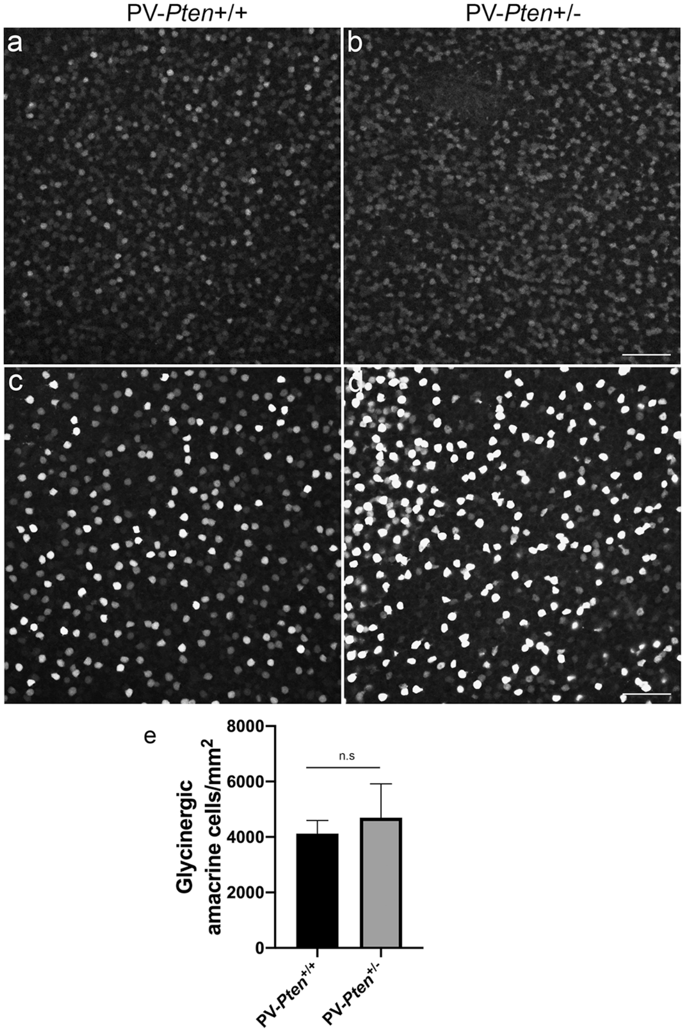

S. Figure 2. Gly immunoreactivity in PV-*Pten*$^{+/+}$ and PV-*Pten*$^{+/–}$ whole-mounted retinas. (a) Gly immunoreactivity in bipolar cells in PV-*Pten*$^{+/+}$ whole-mounted retinas. (b) Gly immunoreactivity in bipolar cells in PV-*Pten*$^{+/–}$ whole-mounted retinas. (c) Gly immunoreactivity in amacrine cells in PV-*Pten*$^{+/+}$ whole-mounted retinas. (d) Gly immunoreactivity in amacrine cells in PV-*Pten*$^{+/–}$ whole-mounted retinas. (e) Histogram indicating the mean (± SEM) of Gly positive amacrine cells in PV-

Pten$^{+/+}$ (n=4 retinas) and PV-*Pten*$^{+/-}$ retinas (n= 3 retinas, $P > 0.05$, Mann-Whitney test). z-step = 1 µm. 8–10 optical sections were compressed for viewing. Scale bar = 50 µm.

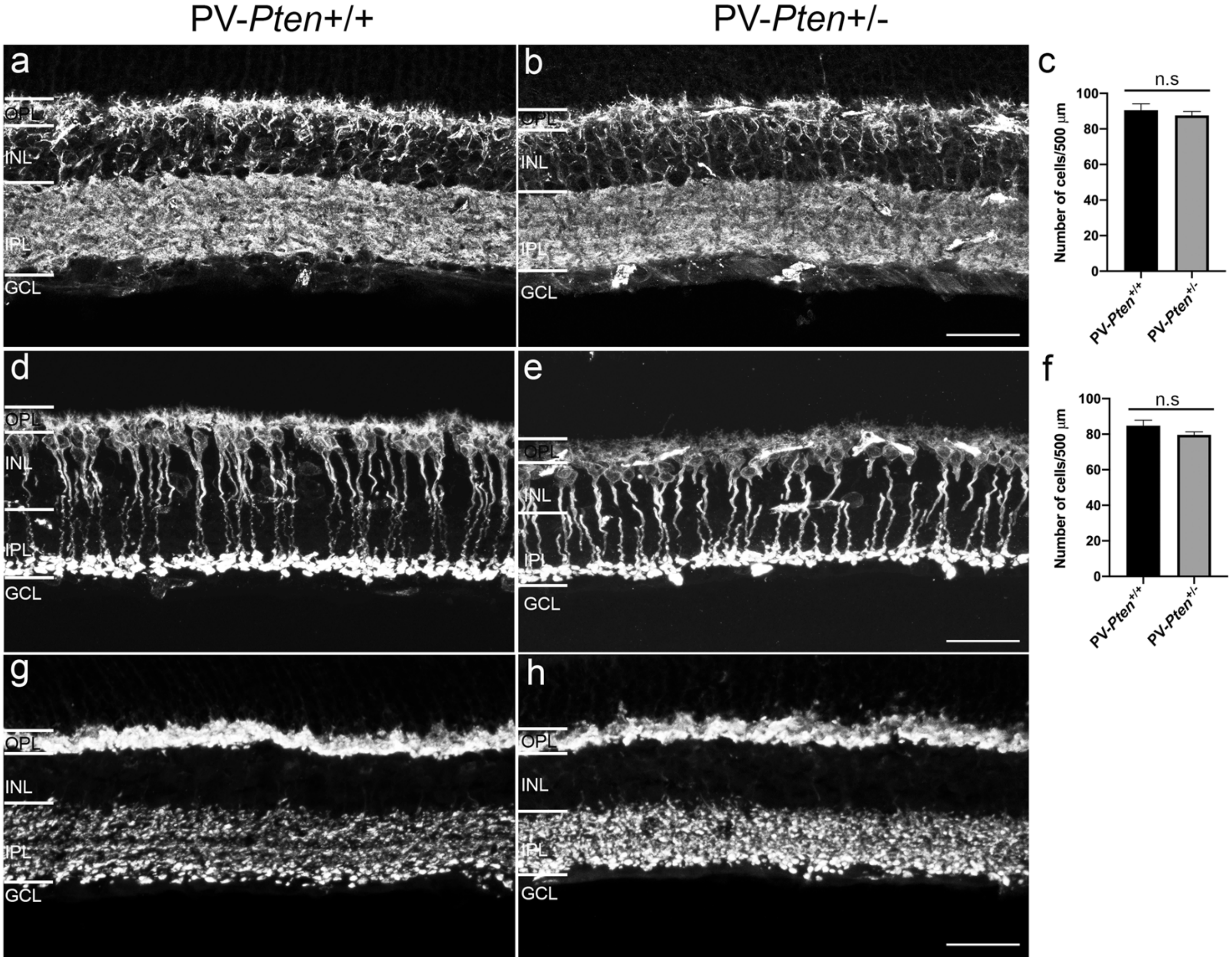

S. Figure 3. Goα, PKC, and VGluT1 immunoreactivity in PV-*Pten*$^{+/+}$ and PV-*Pten*$^{+/-}$ retinal sections. (a) Goα immunoreactivity in ON-type bipolar cells in PV-*Pten*$^{+/+}$ retinas. (b) Goα immunoreactivity in ON-type bipolar cells in PV-*Pten*$^{+/-}$ retinas. (c) Histogram indicating the mean (± SEM) of Goα positive cells per 500 µm in PV-*Pten*$^{+/+}$ (n=2 retinas) and PV-*Pten*$^{+/-}$ retinas (n=2 retinas, $P > 0.05$, Mann-Whitney test). (d) PKC immunoreactivity in rod-type bipolar cells in PV-*Pten*$^{+/+}$ retinas. (e) PKC immunoreactivity in rod-type bipolar cells in PV-*Pten*$^{+/-}$ retinas. (f) Histogram indicating the mean (± SEM) of PKC positive cells per 500 µm PV-*Pten*$^{+/+}$ (n=2 retinas) and PV-*Pten*$^{+/-}$ retinas (n=2 retinas, $P > 0.05$, Mann-Whitney test). (g) VGluT1 immunoreactivity in bipolar cell and photoreceptor terminals in PV-*Pten*$^{+/+}$ retinas. (h) VGluT1 immunoreactivity in bipolar cell and photoreceptor terminals in PV-*Pten*$^{+/-}$ retinas. z-step = 1 µm; 3–4 optical sections were compressed for viewing. OPL, Outer plexiform layer; INL, Inner nuclear layer; IPL, Inner plexiform layer; GCL, Ganglion cell layer. Scale bar = 50 µm.
